# *PIN1a*-mediated auxin release from rootstock cotyledon contributes to healing in watermelon as revealed by seeds soaking-VIGS and cotyledon grafting

**DOI:** 10.1093/hr/uhae329

**Published:** 2024-11-26

**Authors:** Xiao Wang, Mu Xiong, Jianuo Xu, Ting Zhang, Akebaierjiang Kadeer, Zhilong Bie, Michitaka Notaguchi, Yuan Huang

**Affiliations:** National Key Laboratory for Germplasm Innovation and Utilization of Horticultural Crops, College of Horticulture and Forestry Sciences, Huazhong Agricultural University, 430070 Wuhan, China; National Key Laboratory for Germplasm Innovation and Utilization of Horticultural Crops, College of Horticulture and Forestry Sciences, Huazhong Agricultural University, 430070 Wuhan, China; Graduate School of Bioagricultural Sciences, Nagoya University, Aichi 464-8601, Japan; National Key Laboratory for Germplasm Innovation and Utilization of Horticultural Crops, College of Horticulture and Forestry Sciences, Huazhong Agricultural University, 430070 Wuhan, China; National Key Laboratory for Germplasm Innovation and Utilization of Horticultural Crops, College of Horticulture and Forestry Sciences, Huazhong Agricultural University, 430070 Wuhan, China; National Key Laboratory for Germplasm Innovation and Utilization of Horticultural Crops, College of Horticulture and Forestry Sciences, Huazhong Agricultural University, 430070 Wuhan, China; National Key Laboratory for Germplasm Innovation and Utilization of Horticultural Crops, College of Horticulture and Forestry Sciences, Huazhong Agricultural University, 430070 Wuhan, China; National Key Laboratory for Germplasm Innovation and Utilization of Horticultural Crops, College of Horticulture and Forestry Sciences, Huazhong Agricultural University, 430070 Wuhan, China; Graduate School of Bioagricultural Sciences, Nagoya University, Aichi 464-8601, Japan; National Key Laboratory for Germplasm Innovation and Utilization of Horticultural Crops, College of Horticulture and Forestry Sciences, Huazhong Agricultural University, 430070 Wuhan, China; Shenzhen Institute of Nutrition and Health, Huazhong Agricultural University, Shenzhen 518100, China; Shenzhen Branch, Guangdong Laboratory for Lingnan Modern Agriculture, Genome Analysis Laboratory of the Ministry of Agriculture, Agricultural Genomics Institute at Shenzhen, Chinese Academy of Agricultural Sciences, Shenzhen 518100, China

## Abstract

Grafting is a propagation method extensively utilized in cucurbits. However, the mechanisms underlying graft healing remain poorly understood. This study employed self-grafted watermelon plants to investigate how rootstock cotyledon affects healing. The complete removal of rootstock cotyledons significantly hindered scion growth, as evidenced by reductions in scion fresh weight and the area of true leaves. Physiological assessments revealed reduced callus formation, weaker adhesion forces, a more pronounced necrotic layer, and decreased rates of xylem and phloem reconnection at the graft junction when rootstock cotyledons were completely removed. Additionally, auxin levels at the rootstock graft junction notably decreased following cotyledon removal. In contrast, the exogenous application of indole-3-acetic acid (IAA) notably enhanced graft healing. Moreover, gene expression analysis of the *PIN* auxin efflux carriers in the rootstock cotyledons indicated significant activation of *ClPIN1a* postgrafting. Furthermore, we developed an improved Virus-Induced Gene Silencing (VIGS) system for cucurbits using seeds soaking method. This method achieved an infection success rate of 83% with 60%–75% gene silencing efficiency, compared to the 37% success rate with 40%–60% efficiency seen with traditional cotyledon infection. Combining our novel VIGS approach with cotyledon grafting techniques, we demonstrated that rootstock cotyledons regulate callus formation through *ClPIN1a*-mediated endogenous auxin release, thus facilitating graft union development. These findings suggest potential strategies for enhancing watermelon graft healing by manipulating rootstock cotyledons.

## Introduction

Grafting is a traditional agricultural technique with historical references dating back to the first century BC, as mentioned in the ancient Chinese agricultural text ‘Fan Sheng-chih shu’ [1]. In modern agriculture, the grafting of cucurbit crops is extensively practiced to enhance resistance to biotic and abiotic stresses and improve resource utilization efficiency [2, 3]. Traditional watermelon grafting methods require the retention of at least one rootstock cotyledon during the healing period to ensure graft success [4]. The removal of rootstock cotyledons in watermelon has been associated with a marked decrease in graft survival rates compared to those with at least one cotyledon [5, 6]. Research on cucumbers has demonstrated that cotyledon removal at the graft site can inhibit cell division at the incision site [7] and delay the formation of vascular bridges essential for grafting success [8]. Additionally, the large cotyledons characteristic of cucurbit crops can create excessive local humidity during the healing period, fostering fungal infections. Although removing rootstock cotyledons can reduce the risk of fungal diseases, it also lowers the survival rate of grafted plants.

Cotyledons are vital organs in young seedlings, storing essential nutrients and hormones crucial for early plant development. They play a pivotal role in hormone release prior to tissue reunion and grafting. Specifically, gibberellin (GA) is fundamental for tissue reunion in the hypocotyl cortex of tomato and cucumber seedlings, with cotyledons significantly influencing GA levels in the hypocotyls. Local application of GA biosynthesis inhibitors to cotyledons can effectively suppress cell division during tissue reunion, whereas applying GA can reverse this inhibition [7]. In *Arabidopsis*, cotyledon-derived auxin aids tissue reunion in a cell type-specific manner by regulating cell proliferation in vascular tissues and promoting the expansion of cortex cells through GA biosynthesis during the reunion process [9]. Elevated temperatures increase auxin levels in the scion's cotyledon, facilitating its movement to the graft junction where it degrades BODENLOS (BDL), a transcriptional corepressor of the AUX/IAA family, and activates auxin response factors (ARFs), thereby promoting phloem reconnection [10]. Additionally, studies have shown that etiolated cucumber scions delay vascular reconnection and reduce graft survival; however, these effects can be mitigated by the exogenous application of glucose, highlighting the positive role of sugars in graft healing [11].

Auxin, primarily produced in young leaves, is released to the wound site to regulate tissue repair [10, 12, 13]. It plays a crucial role in tissue regeneration and the reconnection of vascular tissues [14, 15], and is involved in the regulation of xylem development and cambium growth [16, 17]. Auxin promotes graft healing by inducing callus formation [18, 19], as observed in tobacco, where overexpression of the auxin biosynthetic gene *iaaM* in rootstocks increases endogenous auxin levels, accelerating callus development to bridge the gaps between the scion and rootstock [20]. Application of 2,3,5-triiodobenzoic acid (TIBA, an auxin transport inhibitor) in *Arabidopsis* impedes vascular cell growth at the graft site [12]. In Chinese cabbage, auxin produced in cotyledons is also known to regulate hypocotyl elongation [21].

Currently, the specific mechanisms by which auxin, derived from the cotyledons of watermelon rootstocks, facilitates graft healing remain poorly understood. This gap in knowledge presents an opportunity to deepen our understanding of watermelon grafting processes. Cotyledons are important sources of auxin in developing plants [22]. It is transported to the plant base by PIN-formed (PIN) proteins [23, 24]. These efflux carriers are important for the regeneration of vascular tissues. In *Arabidopsis* inflorescence stems, increased auxin levels near the incision site activate *PIN1*-mediated tissue polarity rearrangement, facilitating the formation of new circular vessels and vessel strands within the callus region [25, 26]. Mutations in *PIN1* or the application of the auxin transport inhibitor TIBA inhibit cell proliferation in the pith tissue during tissue reunion [12]. Furthermore, the directional movement of auxin mediated by PIN proteins promotes the aggregation of vascular tissues, enhancing graft healing efficiency [27].

In *Arabidopsis*, *AtPIN1* is critical for cotyledon development, facilitating the long-distance transport of growth hormones along the plant [28]. Grafting-induced stress affects the expression of *PIN* genes, with *AtPIN1* playing a significant role in the grafting process of *Arabidopsis* [29]. Similar induction of *PIN* gene expression following grafting has been observed in watermelon [30]. Additionally, *PIN1*-mediated auxin transport is crucial for vascular differentiation. For example, elevated expression of *PIN1* has been noted in the damaged vascular stems of peas, suggesting its vital role in vascular healing [26]. Moreover, in hickory, auxin has been shown to facilitate graft healing, whereas the auxin transport inhibitor NPA impedes this process. This inhibition is accompanied by a significant upregulation of *CcPIN1a* and *CcPIN1b* postgrafting, suggesting their involvement in graft healing regulation [31].

Due to the labor-intensive and time-consuming nature of genetic transformation in cucurbit crops, the application of gene editing technologies is limited in these species. Virus-induced gene silencing (VIGS) has emerged as an essential and rapid method for gene function analysis, particularly in species that lack a stable genetic transformation system [32, 33]. As a reverse genetics tool, VIGS allows for the reduction of gene transcription levels in plants, facilitating rapid functional analysis [34–36]. To date, various inoculation methods such as leaf injection, spray inoculation, agricultural irrigation, and vacuum infiltration have been established to study gene function in plants [37–40]. However, these methods have rarely been applied at the early stages of plant development, from seed germination to early seedling morphogenesis. Since cucurbit plants are typically grafted at the young seedling stage, traditional leaf injection techniques, which achieve gene silencing after the optimal grafting age, are not ideal for the gene functional study of graft healing.

Here, we enhanced the infection method by using seeds soaking rather than the traditional cotyledon injection approach. Additionally, we assessed the infection efficacy across different solution concentrations to establish a VIGS system suitable for the functional verification of genes in cucurbit grafting research. Moreover, we assessed physiological indices and conducted histological observations on self-grafted watermelon seedlings, both with and without rootstock cotyledons. We analyzed changes in auxin levels at the grafting incisions of the scion and rootstock, and applied exogenous indole-3-acetic acid (IAA) to grafted seedlings after the removal of rootstock cotyledons to monitor their growth. Furthermore, the role of *ClPIN1a* from rootstock cotyledons was investigated using cotyledon-grafting technology in conjunction with the optimized VIGS system to elucidate the molecular mechanisms by which rootstock cotyledons influence graft healing. This research reveals how *ClPIN1a* regulates auxin in rootstock cotyledons to improve graft healing, enhancing both theoretical knowledge and practical techniques for watermelon grafting.

## Results

### Removal of rootstock cotyledons delays scion growth and graft healing

**Figure 1:**
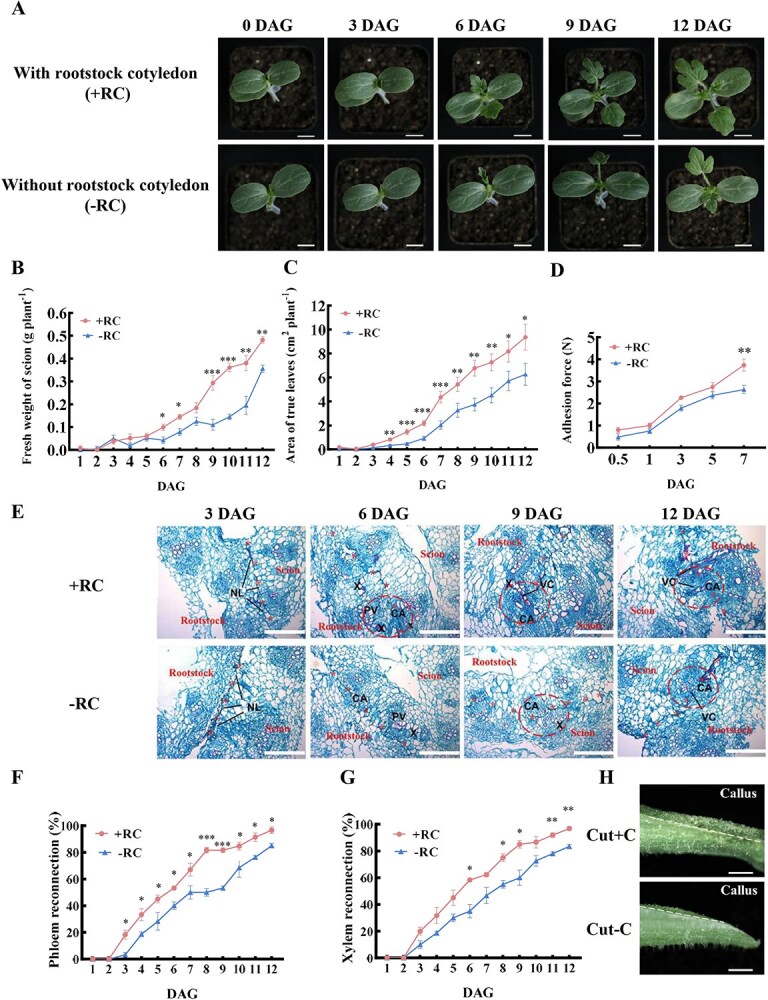
Role of rootstock cotyledons in watermelon grafting. **A**: Growth comparison of self-grafted watermelon with (+RC) and without (–RC) rootstock cotyledons during the graft healing period. DAG: days after grafting. Scale bar: 2 cm. **B**: Fresh weight of the scion in self-grafted watermelon with and without rootstock cotyledons. Fifteen plants per replicate were selected from each grafting type at various time points. Data are shown as mean ± SE (*n* = 3), with statistical significance indicated (^*^P < 0.05, ^**^P < 0.01, ^***^P < 0.001, Student’s *t*-test). **C**: Leaf area of self-grafted watermelon with and without rootstock cotyledons. Data collection and statistics as in **B**. **D**: Adhesive force between the scion and rootstock in self-grafted watermelon with and without rootstock cotyledons. Data collection and statistics as in **B**. **E**. Paraffin sections showing the graft junction in self-grafted watermelon with and without rootstock cotyledons. NL: necrotic layer, VC: vascular bundle reconnection, CA: callus formation, X: new xylem elements, and PV: original vascular bundles. Asterisks highlight the grafting surface, and the dotted lines indicate the graft healing interface. Scale bar: 300 μm. **F**: Phloem reconnection in self-grafted watermelon with and without rootstock cotyledons. Fifteen plants per replicate were selected from each graft combination at various time points. Data are shown as mean ± SE (*n* = 3), with significance levels indicated (^*^P < 0.05, ^***^P < 0.001, Student’s *t*-test). **G**: Xylem reconnection in self-grafted watermelon with and without rootstock cotyledons, with data collection and statistical analysis as in **F**. **H**: Callus formation at the incision site on the sixth day after grafting in cut rootstock hypocotyls with (Cut+C) and without (Cut−C) rootstock cotyledons. The cut surface is marked with dotted lines. Scale bar: 3 mm.

Watermelon seedlings were grafted with and without rootstock cotyledons. Compared to the plants with a rootstock cotyledon, those without showed significantly weaker growth starting from 6 days after grafting (DAG) (Fig. 1a). We continuously monitored the true leaf area, fresh weight, and physical adhesion force of the scion. These measurements indicated that the healing process for grafts without rootstock cotyledons was delayed by ~3 days relative to those with cotyledons. Specifically, the fresh weight of the scion at 6 DAG was equivalent to that observed at 9 DAG (Fig. 1b), and the true leaf area of the scion at 7 DAG was comparable to that at 10 DAG (Fig. 1c). Additionally, the adhesion force between the rootstock and scion was stronger in grafted plants with rootstock cotyledons compared to those without (Fig. 1d). Overall, the growth trends suggest that grafted plants with rootstock cotyledons outperformed those without.

To investigate graft union formation, we conducted paraffin section observations. At 3 DAG, both graft combinations exhibited noticeable necrotic layers at the graft boundaries (Fig. 1e). By 6 DAG, proliferated cells filled the gaps caused by incisions between the scion and rootstock, forming callus. In grafts with rootstock cotyledons, these callus cells were compressed and fused toward the healing surface, facilitating the gradual degradation of necrotic layers (Fig. 1e). In contrast, grafts without rootstock cotyledons showed less efficient degradation of these layers (Fig. 1e). Additionally, more xylem elements were observed at the graft boundary and formed xylem bridges in grafts with rootstock cotyledons than in those without (Fig. 1e).

For further analysis of vascular reconnection during graft union development, esculin and acid fuchsin were applied to the scion and rootstock, respectively, to monitor the fluorescence signal in both parts. The rate of xylem and phloem reconnection in grafts with rootstock cotyledons reached 50% by 5 DAG, whereas in grafts without rootstock cotyledons, this process was delayed by 2 days, aligning with previous growth data (Fig. 1f, 1g). Additional experiments on cut hypocotyls demonstrated less callus formation at the incision site 6 days postcutting in samples without rootstock cotyledons compared to those with cotyledons (Fig. 1h). Overall, the absence of rootstock cotyledons in grafted plants impairs callus differentiation at the graft healing interface and subsequently delays scion growth.

### Rootstock cotyledon-derived IAA delays graft healing

**Figure 2:**
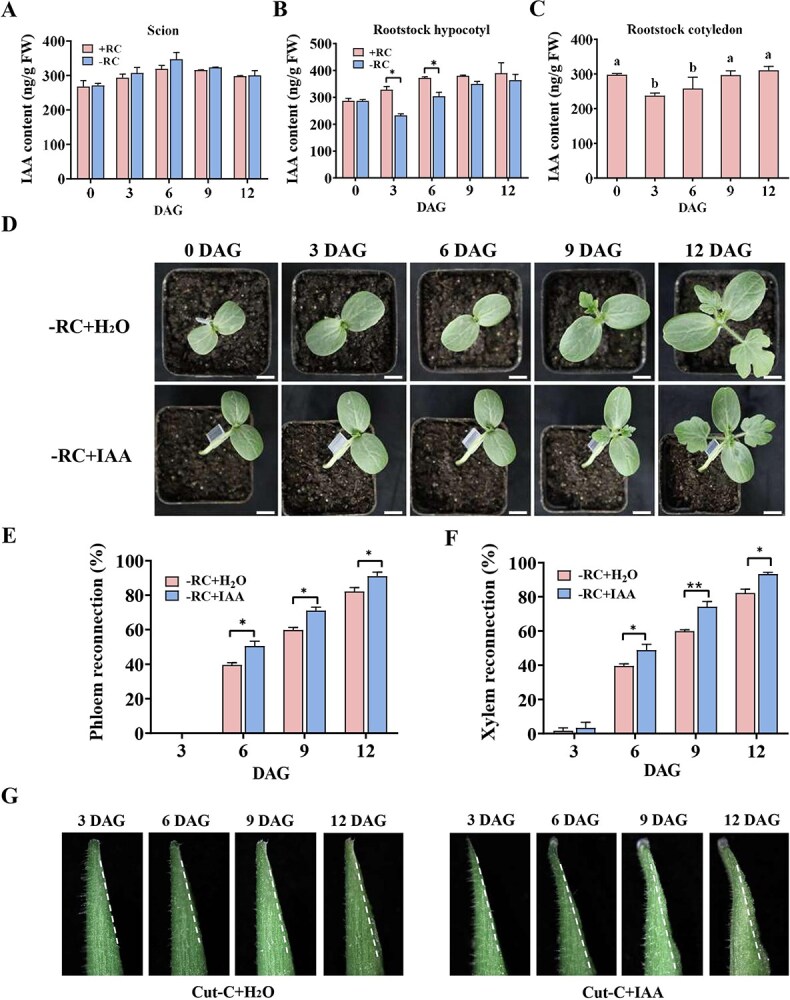
IAA content and its effect on graft union formation. **A**: Endogenous auxin content in the scion at the graft junction during the graft healing period. Five plants per replicate were selected from each graft combination at various time points. Data are expressed as mean ± SE (*n* = 4). **B**: Endogenous auxin content in the rootstock at the graft junction during the graft healing period. Five plants per replicate were selected from each graft combination at various time points. Data are expressed as mean ± SE (*n* = 4) (^*^P < 0.05, Student’s *t*-test). **C**: Endogenous auxin content in rootstock cotyledons during the graft healing period. Five plants per replicate were selected from each graft combination at various time points. Data are expressed as mean ± SE (*n* = 4). Different letters indicate statistically significant differences among groups (Tukey’s honestly significant difference test, *P* < 0.05). **D**: Growth of self-grafted watermelon with and without rootstock cotyledons under exogenous auxin treatment. Scale bar: 2 cm. **E**: Phloem reconnection rate in self-grafted watermelon with and without rootstock cotyledons under exogenous auxin treatment. Fifteen plants per replicate were selected from each graft combination at various time points. Data are expressed as mean ± SE (*n* = 3) (^*^P < 0.05, Student’s *t*-test). **F**: Xylem reconnection rate in self-grafted watermelon with and without rootstock cotyledons under exogenous auxin treatment. Fifteen plants per replicate were selected from each graft combination at various time points. Data are expressed as mean ± SE (*n* = 3) (^*^P < 0.05, ^**^P < 0.01, Student’s *t*-test). **G**: Callus cells at the cut site of the hypocotyl with and without cotyledons under exogenous auxin treatment. Scale bar: 2 mm, Cut−C + H_2_O: Grafts without rootstock cotyledons treated with water. Cut−C + IAA: Grafts without rootstock cotyledons treated with exogenous auxin.

To further investigate the influence of rootstock cotyledons on graft healing, we analyzed endogenous auxin (IAA) levels at the graft junction across the scion, rootstock, and rootstock cotyledon. Within 6 days postgrafting, the scion's endogenous IAA levels initially increased, followed by a gradual decline from Day 6 onward, regardless of the presence or absence of rootstock cotyledons (Fig. 2a). This pattern may be due to vascular bundle obstruction caused by injury at the early stages of graft healing, which results in the accumulation of auxin at the graft interface. Upon the reestablishment of vascular connections, the accumulated auxin redistributes to the rootstock, and its levels decrease. Notably, auxin contents in scions grafted with or without rootstock cotyledons showed no significant differences, suggesting that removing the rootstock cotyledons does not affect auxin levels in the scion. By contrast, in the rootstock, IAA content continuously increased during the healing process when the rootstock cotyledon was present (Fig. 2b). In grafts without rootstock cotyledons, however, IAA levels dropped significantly by Day 3 and remained lower on Days 3 and 6 compared to those with cotyledons (Fig. 2b). Additionally, IAA levels in the cotyledon decreased by 3 DAG (Fig. 2c). These findings suggest that auxin or its derivatives are transported from the cotyledon to the graft region in the rootstock after grafting, increasing auxin levels in the rootstock. Therefore, the auxin found in rootstocks primarily derives from the cotyledons of the rootstock, and the auxin released from these cotyledons significantly impacts the overall auxin levels within the rootstocks.

The exogenous application of auxin to the leaves of scion without rootstock cotyledons during the graft healing period significantly enhanced plant growth and markedly improved the reconnection rates of xylem and phloem (Fig. 2d–f). Additionally, applying auxin to the cut hypocotyl enhanced callus formation at the incision site (Fig. 2g). As previously discussed, this exogenous auxin application effectively alleviated the delayed healing associated with the removal of rootstock cotyledons.

### A cotyledon grafting method and an efficient VIGS system suitable for cucurbit plants

**Figure 3:**
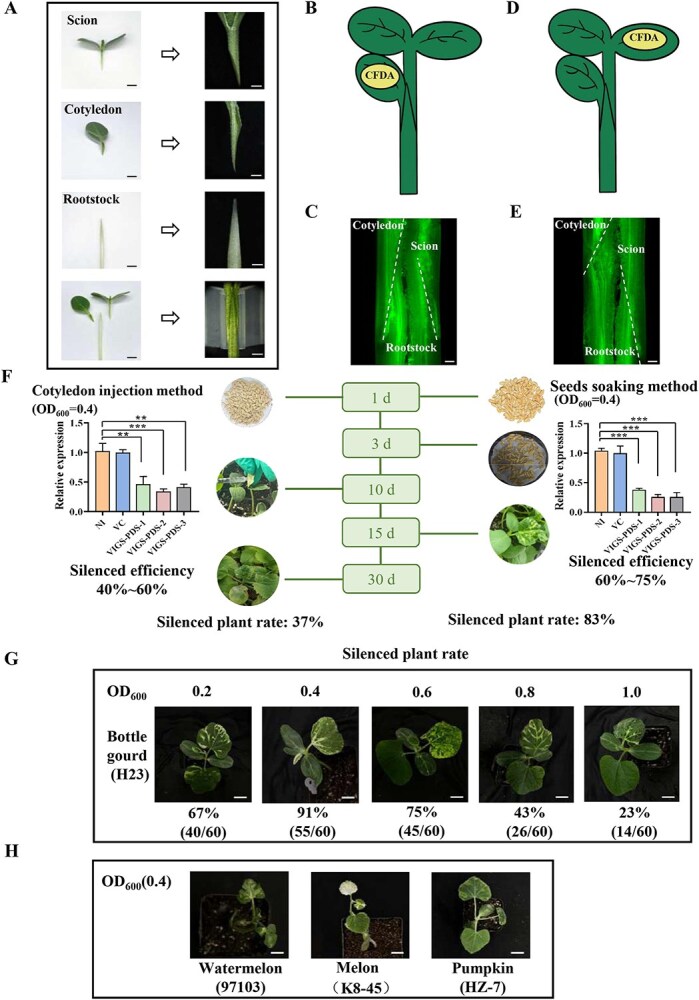
A cotyledon grafting method and an improved VIGS system in cucurbit. **A**: Illustration of the cotyledon grafting process. **B**: Application of CFDA to the grafted rootstock cotyledons at 12 DAG. **C**: Distribution of CFDA signal in the scion, rootstock, and grafted cotyledon following its application to the rootstock cotyledon. Scale bar: 500 μm. **D**: Application of CFDA to the scion at 12 DAG. **E**: Distribution of CFDA signal in the scion, rootstock, and grafted cotyledon after CFDA application to the scion. Bar: 500 μm. **F**: Comparative analysis of VIGS efficacy between the traditional cotyledon infection method and the seeds soaking approach in melon. Silencing rates and efficiencies were assessed 3 weeks postcotyledon injection (4 weeks after sowing), which revealed 11 albino plants out of 30 sampled, and 2 weeks after sowing by the seed-soaking method, which identified 25 albino plants out of 30 sampled. NI: noninoculated. VC: vector control inoculated. *PDS*: *Phytoene Desaturase* gene. Data are expressed as mean ± SE (*n* = 3) (^**^P < 0.01, ^***^P < 0.001, Student’s *t*-test). **G**: Effect of inoculation concentration on silencing efficiency. **H**: Gene silencing in other cucurbit crops using the improved VIGS system at an OD_600_ value of 0.4. Scale bar: 2 cm.

To investigate the role of auxin from rootstock cotyledons in graft healing, we developed a cotyledon grafting method and established an efficient VIGS system using a seed-soaking approach in cucurbits (Fig. 3). In our technique, the scion, rootstock, and cotyledon are grafted simultaneously (Fig. 3a). This method achieved a graft survival rate of 85%. At 12 DAG, 5(6)-carboxyfluorescein diacetate (CFDA) was applied to the cotyledon grafted onto the rootstock, and its signal was detected in both the scion and rootstock tissues after 1 h (Fig. 3b, c). Similarly, when CFDA was applied to the scion cotyledon, its presence was detected in both the rootstock and the grafted cotyledon after 1 h (Fig. 3d, e). These results demonstrate the reestablishment of vascular transport connections between the rootstock, scion, and grafted rootstock cotyledon. By treating the cotyledon as a separate part in the grafted plant, this method facilitates a more detailed study of cotyledon function.

For the VIGS system in cucurbit grafting studies, we initially tested the traditional VIGS approach in melon. Using the *Phytoene Desaturase* (*PDS*) gene as the indicator, photobleaching appeared at 35 days after traditional cotyledon infection, with only 37% of the plants successfully infected and achieving a gene silencing efficiency of 40%–60% (Fig. 3f). However, this timing was suboptimal for cucurbit grafting studies due to the age of the plants and potential adverse effects from damaged cotyledons. To obtain VIGS plants at an appropriate age and with intact cotyledons for grafting, we soaked germinated seeds in an *Agrobacterium* inoculation solution for 1 day before sowing. Two weeks postsowing, photobleaching appeared on the first true leaves of melon, with an infection success rate of 83% and silencing efficiency between 60% and 75% (Fig. 3f). Additionally, we established a concentration gradient for the *Agrobacterium* inoculation solution (OD_600_ = 0.2, 0.4, 0.6, 0.8, and 1.0) and used it to infect germinated bottle gourd seeds through soaking. Three weeks postsowing, we evaluated the number of plants exhibiting bleaching and calculated the VIGS silencing rates. The highest silencing rate was observed at an OD_600_ of 0.4, reaching 91%, followed by rates of 75% and 67% at OD_600_ = 0.6 and 0.2, respectively. The lowest silencing rate was only 23% at an OD_600_ of 1.0 (Fig. 3g), indicating that a higher concentration of *Agrobacterium* inoculation solution does not necessarily improve results, with the optimal concentration being OD_600_ = 0.4 for bottle gourd. Subsequently, we applied this seed-soaking method to other cucurbit crops, such as watermelon and pumpkin, to evaluate our improved VIGS system. By infecting germinated seeds with the *Agrobacterium* solution at OD_600_ = 0.4, successful gene silencing was also achieved in pumpkins and watermelons (Fig. 3h).

### Impact of *ClPIN1a*-silencing on auxin transport and graft healing in watermelon cotyledons

**Figure 4:**
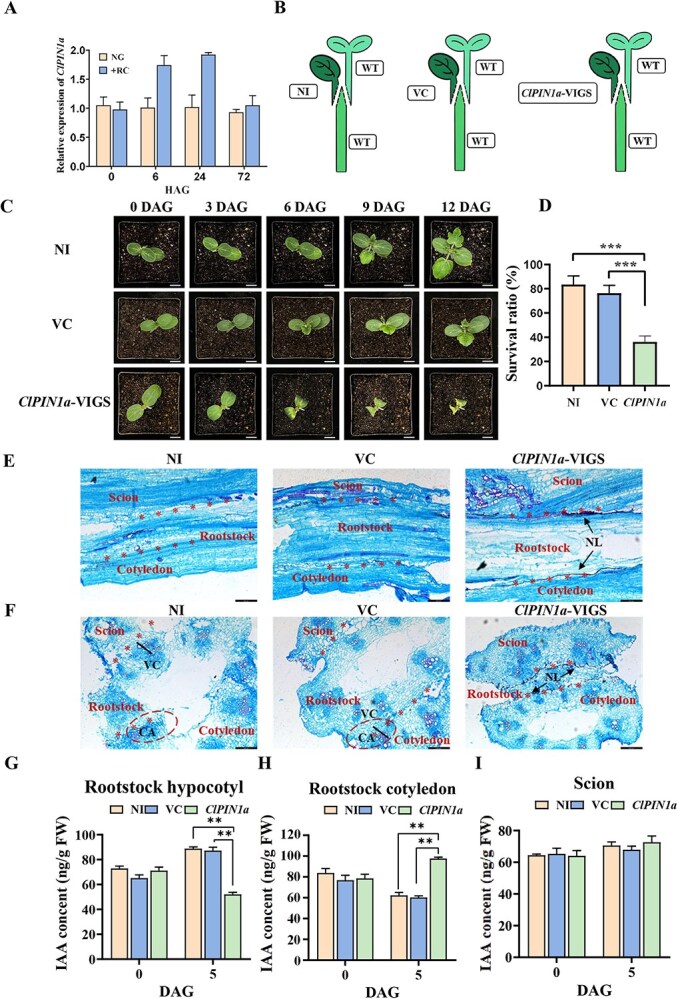
Silencing of *ClPIN1a* in grafted cotyledons delays graft union formation. **A**: Expression of *ClPIN1a* in rootstock cotyledons during the graft healing period. NG: nongrafted; +RC: grafted with rootstock cotyledons. **B**: Grafts with non-infected cotyledons (NI), empty vector-infected cotyledons (VC), and *ClPIN1a*-VIGS-infected cotyledons. WT: wild type. **C**: Plant growth of NI, VC, and *ClPIN1a*-VIGS grafts during the healing period. Scale bar: 2 cm. **D**: Survival rate of NI, VC, and *ClPIN1a*-VIGS grafts at 12 DAG. Twenty-four plants per replicate were selected from each graft combination at various time points. Data are expressed as mean ± SE (*n* = 3) (^***^P < 0.001, Student’s *t*-test). **E**: Longitudinal sections of paraffin-embedded tissues showing the formation and differentiation of healing tissues at 7 DAG. NL: necrotic layer; VC: vascular bundle reconnection; CA: callus; X: new xylem elements. Asterisks indicate the grafting surface. Scale bar: 500 μm. **F**: Cross-sections of paraffin-embedded tissues showing the formation and differentiation of healing tissues at 7 DAG. The dotted lines indicate the graft healing interface. **G**: Auxin content in the rootstock of *ClPIN1a*-VIGS cotyledon grafted plants. **H**: Auxin contents in the rootstock cotyledon of *ClPIN1a*-VIGS grafted plants. **I**: Auxin content in the scion of *ClPIN1a*-VIGS grafted plants. Five plants per replicate were selected from each graft combination at different time points. Data are expressed as mean ± SE (*n* = 4) (^**^P < 0.01, Student’s *t*-test).

To investigate the role of auxin transport in rootstock cotyledons, we analyzed the *PIN* family genes in watermelon (*Citrullus lanatus*). Using intact plants as controls, we determined the expression of *ClPIN* family genes in rootstock cotyledons via Real-Time Quantitative Reverse Transcription PCR (qRT-PCR). The results showed that 13 *ClPIN* genes were upregulated after grafting (Fig. S1), with *ClPIN1a* showing the highest increase in expression at 6 and 24 h after grafting (HAG) (Fig. 4a). To further investigate the role of *ClPIN1a* in graft healing, we performed cotyledon grafting using *ClPIN1a-VIGS* plants with wild-type scions and rootstocks (Fig. 4b). The survival rate of grafts with *ClPIN1a-VIGS* cotyledons was significantly lower compared to those with noninfected (NI) and empty vector-infected (VC) cotyledons (Fig. 4c, 4d). Anatomical observations indicated that while callus formation and vascular reconnection occurred without a necrotic layer in NI and VC grafts, the graft junctions of *ClPIN1a-VIGS* combinations exhibited an obvious necrotic layer on both sides at 7 DAG (Fig. 4e, 4f). Furthermore, auxin levels were measured in different plant organs at 5 DAG. Compared to NI and VC, the auxin content in the rootstock cotyledon of *ClPIN1a-VIGS* grafts was significantly higher (Fig. 4h), while it was significantly reduced in the rootstock (Fig. 4g), and there was no significant difference in the scion (Fig. 4i). Together, these results suggest that silencing of the auxin transporter gene *ClPIN1a* in rootstock cotyledons disrupts auxin transport and adversely affects the graft healing process.

## Discussion

### Role of rootstock cotyledon-derived auxin in enhancing graft healing and vascular reconnection in watermelon

The healing process in grafted plants can generally be divided into three stages: formation of a necrotic layer between the rootstock and scion, cell proliferation between the two to form callus, and the subsequent differentiation of the callus into xylem and phloem elements. This differentiation leads to the disappearance of the necrotic layer and connects the vascular bundles [41]. The removal of rootstock cotyledons leads to weaker physical adhesion and reduced callus cell proliferation at the graft junction, which likely impairs early adhesion between the rootstock and scion. Previous research supports this, noting that a reduction in rootstock cotyledons affects cell division and vascular bridge formation at the cut site [7, 8]. Consistent with these findings, our study observed a decrease in callus proliferation and a delay in necrotic layer disappearance and vascular reconnection by 2–3 days in watermelon after rootstock cotyledon removal. Cotyledon-derived auxin in *Arabidopsis* has been shown to promote vascular tissue cell proliferation and cortex cell expansion [9, 10]. Our findings suggest that rootstock cotyledons are an important auxin source during the healing process in watermelon grafts. When cotyledons were removed, auxin levels at the graft junction decreased, leading to fewer callus cells at the incision site. However, the application of exogenous IAA to the cut hypocotyl of cotyledon-removed plants increased callus formation at the incision, indicating that rootstock cotyledon-derived auxin is critical for promoting callus formation and facilitating successful graft healing.

### A cotyledon grafting method and an improved VIGS system suitable for young seedlings

Cotyledon grafting technology serves as an effective tool for investigating the early growth and developmental processes in plants, particularly the long-distance transport interactions between various plant organs. Unlike the smaller cotyledons of *Arabidopsis*, cucurbit cotyledons are larger and more robust, making grafting operations more manageable and thus better suited for studies in cucurbits. Our experiments with watermelon cotyledon grafting have achieved a survival rate of 85% It is crucial to ensure that cuts on both sides of the rootstock are as uniform as possible and to avoid excessive cutting, which can lead to a fragile healing interface, potentially causing failures in the later stages of cotyledon or scion connection. Previous research using cotyledon grafting techniques has illuminated the significant role of cotyledons in flower development [42, 43]. Therefore, establishing a robust watermelon cotyledon grafting technology is essential for advancing our understanding of watermelon cotyledon function. Cucurbitaceae crops serve as an effective model for studying grafting mechanisms. However, challenges arise as some Cucurbitaceae crops lack a stable genetic transformation system, or exhibit very low transformation efficiencies [44]. These limitations significantly impede gene functional studies in cucurbit grafting. For example, Liu *et al.* [45] developed a cotyledon-infection VIGS system in cucumber using the cucumber *green mottle mosaic virus* vector. In cucurbit, however, the numerous veins on the cotyledons complicate the injection of the infection solution, often requiring multiple attempts. This not only damages the cotyledons irreversibly but also adversely affects seedling growth. Furthermore, the older age of successfully gene-silenced seedlings is not ideal for graft survival, making the cotyledon infection method unsuitable for cucurbit grafting studies. Previous studies employed vacuum infiltration to inoculate tomato buds [46]. Inspired by this, we attempted vacuum infiltration on melon ‘Akekekouqi’ seeds, but unfortunately, we did not achieve gene silencing in two trials. Considering the role of roots as active absorption organs that intake substances from the environment into the plant body [47], we optimized the melon VIGS using a seed-soaking infection method. We soaked germinated seeds in the infection solution for 1 day before sowing, and by targeting the *PDS* gene, observed bleaching on the second leaf. We then successfully extended this method to other cucurbit crops, achieving gene silencing in bottle gourd, pumpkins, and watermelons. Notably, the albino phenotype could be observed as early as the first true leaf stage, ~12 days after sowing. Compared with the cotyledon injection method, the optimized seed-soaking VIGS system simplifies the infection process, minimizes physical damage to plants, and demonstrates higher survival rates, silencing rates, and silencing efficiency. The resulting silenced young plants are suitable for studies in graft biology. However, there are some limitations to this system; for instance, watermelon seeds must be peeled to achieve effective silencing, and the silencing efficiency in pumpkins is low. Continuous optimization of this system is necessary to improve its silencing effects.

### *ClPIN1a* mediates auxin transport in rootstock cotyledons to promote graft healing

**Figure 5:**
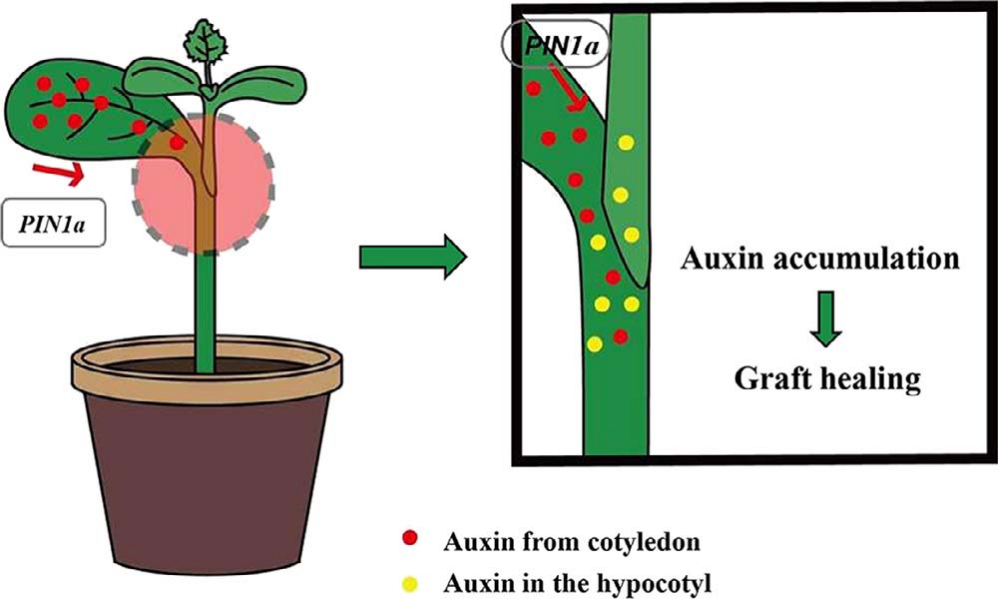
*ClPIN1a*-mediated auxin release from rootstock cotyledons enhances graft healing.

In addition to the endogenous auxin produced in the hypocotyl, significant amounts of auxin are also synthesized in other organs and transported to the incision site [10, 20, 25]. We focused on a key member of the *PIN* family, *ClPIN1a*, during graft union formation. Silencing the *ClPIN1a* gene in cotyledons resulted in increased auxin levels in grafted *ClPIN1a-VIGS* cotyledons compared to the noninoculated (NI) and vector control (VC) groups. This disruption of the auxin transport channel led to a lower survival rate, delayed vascular reconnection, and reduced callus formation, ultimately impeding the graft healing process. By exogenously applying IAA to the *ClPIN1a*-VIGS grafted plants, we observed an increase in graft survival from 32% to 63%, which significantly enhanced the healing process of the grafts (Fig. S3). These findings elucidate the critical role of cotyledon-derived auxin in enhancing graft healing. Grafting is known to induce *PIN* gene expression in both *Arabidopsis* and watermelon, as demonstrated in studies by Melnyk *et al.* [29] and Yu *et al.* [30]. Similarly, increased *PIN1* expression has been observed in damaged pea vascular stems, suggesting a broad role in plant response to injury [26]. In our study, we found that the expression levels of the *ClPIN* gene were elevated postgrafting in watermelon, indicating its involvement in graft healing regulation. This mirrors the patterns observed in hickory, where significant upregulation of *CcPIN1a* in the scion and notable changes in *CcPIN4* expression in the rootstock were noted postgrafting [31], further supporting the regulatory role of *PIN* genes in the grafting process. Our results specifically indicate that *ClPIN1a* expression is most significantly upregulated in the rootstock of watermelon following grafting. This upregulation suggests a critical role for *ClPIN1a* in regulating graft healing, aligning with previous findings where *Arabidopsis AtPIN1* was implicated in regulating growth and development through the long-distance transport of auxin. In our study, silencing the *ClPIN1a* gene in watermelon rootstock cotyledons led to a significant increase in IAA content in cotyledons, whereas IAA content at the graft interface was significantly reduced. This reduction likely resulted from the impaired transport of auxin due to *ClPIN1a* silencing, adversely affecting the graft healing process. These observations are consistent with previous studies, highlighting the essential role of *ClPIN1a* in auxin transport and graft healing dynamics. Therefore, we propose a working model for cotyledon-derived auxin in graft union formation (Fig. 5): during the graft healing process, auxin is transported from the rootstock cotyledon to the healing site by *ClPIN1a*, resulting in its accumulation in the rootstock stems, thereby promoting effective graft healing.

## Materials and methods

### Plant materials and growth conditions

In this study, we used the watermelon cultivar ‘97 103’ (*C. lanatus*) for grafting, cutting, and VIGS experiments. For the VIGS experiments, we utilized the melon cultivar ‘Akekekouqi’ (*Cucumis melo*), pumpkin variety ‘HZ-7’ (*Cucurbita moschata*), and bottle gourd type ‘H23’ (*Lagenaria siceraria*). The plants were grown in a controlled environment room set to a 14 h/10 h day/night cycle, with a light intensity of 300 μmol/m^2^·s^−1^, at a constant temperature of 24°C and 60% relative humidity.

### Determination of growth index of grafted plants

For grafting experiments, 11- and 8-day-old watermelon seedlings were used as the rootstock and the scion, respectively. One cotyledon grafting method was performed as described by Xu *et al.* [48]. The specific methods are as follows: for the rootstock cotyledon (+RC) grafting operation, the rootstock retains one cotyledon, while the other cotyledon is obliquely removed along with the growth point (incision 1 cm). The scion is cut obliquely at the hypocotyl (incision 1 cm), after which the interface is secured. In the without rootstock cotyledon (−RC) grafting operation, both cotyledons and the growth point are obliquely removed from the rootstock (incision 1 cm). The scion is cut in the same manner at the hypocotyl (incision 1 cm), and the interface is then fixed. Light management during the graft healing period includes keeping the grafts in darkness for 0–1 days postgrafting; maintaining a light quantum flux density of ~50 μmol/m^2^·s^−1^ from Days 2–7; and increasing it to ~150 μmol/m^2^·s^−1^ for Days 8–12. The healing photoperiod is maintained at 14 h/10 h day/night. Ventilation management during the graft healing period involves providing ventilation for 0.5 h each morning and afternoon during the first 1–5 days postgrafting; increasing to 1 h each during mornings and afternoons for Days 6–9; ventilating for 2 h each morning and afternoon starting from Day 10; and resuming normal ventilation throughout the day on Days 11–12.

Photographic monitoring of grafted plant growth: for each watermelon self-grafted combination, 15 grafted plants were randomly selected and numbered under both treatments—with and without rootstock cotyledons. These plants were photographed from a top-down perspective at each time point (0, 3, 6, 9, and 12 days after grafting) to monitor their growth.

Determination of scion fresh weight and leaf area: during the graft healing period, five grafted plants per replicate were selected at each time point to measure the scion fresh weight. For leaf area, five grafted plants per replicate were analyzed throughout the entire graft healing period. Each leaf from the grafted plants was individually photographed. ImageJ software (available at https://imagej.nih.gov/ij/) was utilized to calculate the area of each leaf and to compile the total true leaf area for each plant.

Determination of adhesion force in the healing section: the adhesion force between the scions of self-grafted watermelon plants was measured at 12 h, and at 1, 3, 5, and 7 days postgrafting using a tensiometer (ZTS-5 N). At each time point, 15 grafted seedlings were selected for assessment. The procedure was as follows: the tensiometer was positioned horizontally on a table. The anvil part of a grafted seedling was clamped using a small clip, which was then attached to the tensiometer's hook. After removing the grafting clip, the end of the scion was pulled horizontally until it detached, and the tensiometer value was recorded.

### Microscopic analysis of tissue differentiation in healing watermelon plants

Watermelon plants at the one true leaf stage were selected for this study. Two cotyledons were removed using a razor blade at a 45-degree oblique angle at the base of the cotyledon, without grafting. As a control, nongrafted plants with a single cotyledon retained were cultured in the same graft healing environment. Six days after these treatments, ~1-cm stem segments were excised from the wound sites. The differentiation of the healing tissues was then examined under the bright-field conditions of a stereo fluorescence microscope, model M205FA (Leica, Germany).

### Anatomical observation

The collected samples were initially fixed in 70% FAA fixation solution for 24 h, then stored in 70% ethanol at 4°C. We employed the paraffin sectioning method detailed by Xiong *et al.* [49]. Specifically, samples were vertically sectioned to 10 μm using a rotary microtome (Leica RM2255, Leica, Germany). The sections were dewaxed, rehydrated, and stained with 1% safranin for 2 min. Following dehydration, sections were counterstained with 1% fast green in 90% alcohol for 15 s, cleaned, and mounted with neutral balata. Imaging was performed using a positive fluorescence microscope (Leica DM6B, Leica, Germany). Additionally, callus of the cut plants was examined using a stereoscopic fluorescence microscope (Leica M205FA, Leica, Germany).

### Phloem and xylem connectivity assays

Phloem and xylem connectivity was measured as described by Xu *et al.* [48]. The process involved the following steps: [23] Preparation of seedlings: grafted watermelon seedlings were selected, and the root matrix was thoroughly washed, retaining ~3 cm of the roots. The roots were then immersed in a 5 mg/ml Acid Fuschin solution. [50] Cotyledon preparation: the wax on the upper epidermis of the scion’s cotyledon was removed using sandpaper. A layer of 2.5 mM EDTA·2Na solution was evenly applied to the surface of the cotyledon. [12] Esculin application: the Esculin solution was well mixed, and 50 μl of a 20 mg/ml concentration was evenly spread over the surface of the scion cotyledon. The cotyledon was then left to stand in darkness for 2 h. [7] Sample sectioning: scion samples were sectioned into 2-mm stem segments from regions >5 mm distant from the graft healing area. Similarly, rootstock samples were sectioned into 2-mm segments from areas <5 mm below the graft healing region [22] Microscopic observation: for xylem connectivity, observations were made under 630 nm (RFP) excitation light. The rootstock stem segment served as a control to determine if the scion stem segment exhibited fluorescence. For phloem connectivity, observations were made under 454 nm (DAPI) excitation light. The scion stem segment served as a control to check for fluorescence in the rootstock stem segment. Reconnection rates were calculated and presented as the percentage of plants showing a fluorescence signal relative to the total number of plants, expressed as (fluorescence signal plants/total plants) × 100%.

### IAA content determination

Scion samples were collected from a 5-mm stem segment above the graft junction, and rootstock samples were taken from a 5-mm stem segment below the graft junction. Cotyledon samples were comprised of 1-cm^2^ sections taken from rootstock cotyledon leaves. At each time point, 20 grafted plants were randomly selected and divided into four replicates, with each replicate containing five seedlings. Samples were immediately placed in liquid nitrogen and subsequently stored at −80°C. Each sample was fully crushed, and 0.1 g of sample was weighed into a 1.5-ml centrifuge tube. Then, 1 ml of 80% methanol that contained 1 mM 2,6-di-tert-butyl-4-methylphenol was added to the centrifuge tube, and the mixture was extracted for 12 h at 4°C in darkness. After centrifugation, the supernatant was collected for IAA content determination using an Auxin Elisa kit (Code: CD-121844-ELISA, Wuhan Chundu Biotechnology Co., Ltd., China).

### Exogenous auxin treatments

Four hours before grafting, watermelon seedlings were evenly sprayed with a 0.1 mM IAA solution. Each spraying continued until droplets were about to drip from surfaces, followed by a pause to allow for absorption before spraying again. Approximately 3 ml of the solution was used per seedling. For the control treatment, seedlings were sprayed with an equivalent volume of distilled water.

### Cotyledon grafting techniques and fluorescent connectivity determination

The cotyledon grafting procedure is detailed as follows: the scion was obliquely cut 1 cm from top to bottom at a 30-degree angle and the cotyledon was obliquely cut 1 cm from top to bottom at a 30-degree angle along the petiole at the base. The rootstock was obliquely cut 1 cm from bottom to top at a 30-degree angle on both the left and right sides of the stem, ensuring that the left and right incisions were as consistent as possible. The cut scions and cotyledons were then attached to the respective left and right incisions on the rootstock and immediately fixed with a grafting clip.

A CFDA stock solution was prepared by diluting to 10 mg/ml with DMSO and stored at −20°C. The working solution was prepared by further diluting the stock with distilled water to 0.5 mg/ml and stored at −20°C. The epidermal wax of the leaves was carefully removed with abrasive paper, ensuring the cotyledons were not damaged. Next, 50 μl of 2.5 mM EDTA-2Na solution was applied to the cotyledons to inhibit healing and prevent CFDA penetration into the tissue, then 150 μl of 0.5 mg/ml CFDA solution was then added. The treated cotyledons were placed in darkness for 1 h to ensure adequate absorption. The GFP fluorescence was captured using a stereo fluorescence microscope to assess connectivity. Management of the graft healing period for cotyledon-grafted watermelon seedlings was maintained in the same manner as the one-cotyledon grafting method, ensuring consistent care and environmental conditions throughout the healing process.

### RNA extraction, cDNA synthesis, and qRT-PCR

The rootstock samples were collected 5 mm below the graft junction, and the nongrafting (NG) samples were collected 5 mm below the base of the cotyledon. Samples were taken at 3, 6, 9, and 12 DAG. A total of 100 mg of plant tissue was collected in a 1.5-ml RNAase-free centrifuge tube, and liquid nitrogen was added to fully grind the tissue with a mortar. RNA was extracted using TransZol (Code: ET101–01, Beijing TransGen Biotech Co., Ltd., China), following the manufacturer's protocol. cDNA was synthesized using the TransScript® One-Step gDNA Removal and cDNA Synthesis SuperMix Kit (Code: AT311–02, Beijing TransGen Biotech Co., Ltd., China), according to the provided protocol.

Primers were designed using Primer-Blast (https/www.ncbi.nlm.nih.gov/tools/primer-blast/). qRT-PCR was prepared using the 2 × TransStart™ TOP Green qPCR SuperMix Kit (Code: AQ131–01, Beijing TransGen Biotech Co., Ltd., China). The reactions were carried out on a QuantStudio 7 Flex Real-Time PCR System (Applied Biosystems, USA), and relative gene expression levels were calculated using the 2^-ΔΔCt^ method. *Actin* was the reference gene and three independent replicates were used.

### Vector construction and *Agrobacterium* infiltration

The vectors *Cucumber Green Mottle Mosaic Virus (CGMMV)* and *CGMMV-PDS213* used in the VIGS experiments were provided by Gu Qinsheng from the Zhengzhou Fruit Research Institute. The *CGMMV* vector was utilized as described in Liu *et al.* [45]. To silence the *ClPIN1a* gene (*Cla97C04G077450*), a 214-bp fragment of the *ClPIN1a* coding sequence was amplified by PCR and cloned into the CGMMV vector (Fig. S2). We employed BamHI for single-enzyme digestion and assembled the vector via homologous recombination. The primer sequences are listed in Table S1. Prior to *Agrobacterium* infiltration, the *Agrobacterium* strain GV3101 that harbored the *CGMMV* vectors was cultured at 28°C in an LB medium supplemented with antibiotics. After 24 h, the *Agrobacterium* cells were harvested and resuspended in an infiltration buffer that contained 10 mM MgCl_2_, 10 mM MES, and 100 μM acetosyringone.

For cotyledon injection, when the cotyledons of melon plants were flattened (7 days after sowing), ~500 μl of the *Agrobacterium* inoculum solution was injected into the back of each cotyledon using a needle-less syringe. Following injection, the plants were cultured in darkness for 24 h before being transferred to a plant growth room under normal growth conditions.

For seed soaking, germinated cucurbit seeds with slightly opened seed coats and radicles measuring 1–4 cm in length were selected and placed in a 9-cm petri dish. About 40 ml of *Agrobacterium* inoculum solution was poured over the seeds to partially submerge them without completely submerging the radicles. The seeds were incubated in darkness at 20°C–23°C for 24 h and transferred onto sterile filter paper to absorb excess inoculum solution. The seeds were then sown and grown under normal growth conditions.

### Image and data analysis

Statistical analysis and graphical representations were conducted using GraphPad Prism 8. Significant differences among the groups were determined using the Duncan-type new complex range method for multiple comparisons.

## Supplementary Material

Web_Material_uhae329

## Data Availability

Data supporting the findings of this study are available within the paper and its supplementary materials. Additional data are available from the corresponding author upon reasonable request.
