# Supplementary material for: PIN1a-mediated auxin release from rootstock cotyledon contributes to healing in watermelon as revealed by seeds soaking-VIGS and cotyledon grafting
Source: Hortic Res. 2024 Nov 26;12(3):uhae329. doi: 10.1093/hr/uhae329 (PMC11883227; doi:10.1093/hr/uhae329)
Supplement: Web_Material_uhae329 [file web_material_uhae329.zip › Supplementary Table S1.docx]

**Table S1. List of primer sequences for vector construction and qRT-PCR**

| Primer names | Primer sequence（5’—3’） | Note |
| --- | --- | --- |
| CGMMV-*ClPIN1a*-F | CCGTCAGGACTTTACTTAATGGATCCCTCATTGTTCTTGCTGTTCTTGC | Used to construct *ClPIN1a*-VIGS vector |
| CGMMV-*ClPIN1a*-R | TGGTACACATTGATGCTGTTCACGACCTAGACCTATAACTGGATCC |  |
| *ClACT*-F | GAACTTGGCACCTGTCCTGT | Watermelon internal reference primers |
| *ClACT*-R | GAACAGTGCAACAGCCTCAA |  |
| *PDS*-F | TGTGTGGATTACCCTAGACC | Determination of *ClPDS*, *CmPDS*, *CmbPDS* gene expression |
| *PDS*-R | CCAAGCTGCTACCTTTCCAC |  |
| *ClPIN1a*-F | TGCTCGGTATCTGGACAAAGG | Determination of *ClPIN1a* gene expression |
| *ClPIN1a*-R | TGCCGGAAAAATCGCCATAC |  |
| *ClPIN1b*-F | TTGCAGCCCAAGATCATTGC | Determination of *ClPIN1b* gene expression |
| *ClPIN1b*-R | AACTCCTCTAAGCCCAACAGC |  |
| *ClPIN1c*-F | ATCTCCACGAACAATCCCTACG | Determination of *ClPIN1c* gene expression |
| *ClPIN1c*-R | ACGGCAAGAACAGCAAGAAC |  |

(continued table)

| Primer names | Primer sequence（5’—3’） | Note |
| --- | --- | --- |
| *ClPIN1d*-F | ATGTGTTGATGGCGATGGTG | Determination of *ClPIN1d* gene expression |
| *ClPIN1d*-R | TCCTTCTCAGCTTCCGTAATCC |  |
| *ClPIN2a*-F | AATTCATCGCCGCTGATTCC | Determination of *ClPIN2a* gene expression |
| *ClPIN2a*-R | ACGAGAGTGTTGGGAAGAGTG |  |
| *ClPIN2b*-F | CCTTGTTCTTGCCCTGCTTG | Determination of *ClPIN2b* gene expression |
| *ClPIN2b*-R | ACCAATGGCTGTGCTGAGTAG |  |
| *ClPIN2c*-F | ATCGAAATGGAGTGAGGTCGAG | Determination of *ClPIN2c* gene expression |
| *ClPIN2c*-R | GGTTGCTCTTGGGATCATTTGG |  |
| *ClPIN3a*-F | TTGGCTTACGGTTCTGTTCG | Determination of *ClPIN3a* gene expression |
| *ClPIN3a*-R | AAGAGGAACGGCGAAAATGG |  |
| *ClPIN3b*-F | TGCTCCTTTTCGTGTCATCG | Determination of *ClPIN3b* gene expression |
| *ClPIN3b*-R | TCCCTCCTACAATTAGCGTGAC |  |

(continued table)

| Primer names | Primer sequence（5’—3’） | Note |
| --- | --- | --- |
| *ClPIN3c*-F | AAACCGCTTCGTCAGACTTC | Determination of *ClPIN3c* gene expression |
| *ClPIN3c*-R | GCAATTTCCAGAGAGACAGAGC |  |
| *ClPIN5a*-F | TTGGTCCATCACTAGCTTCTCG | Determination of *ClPIN5a* gene expression |
| *ClPIN5a*-R | ATCGTCAGCCAAACAATCGC |  |
| *ClPIN5b*-F | AAAGCGGCCGGAATCATTAG | Determination of *ClPIN5b* gene expression |
| *ClPIN5b*-R | ACTTCCTCCAATTGCTCTGC |  |
| *ClPIN5c*-F | AAAGCGGCCAACACATTAGG | Determination of *ClPIN5c* gene expression |
| *ClPIN5c*-R | TGACCCACACCAAACAACTG |  |
| *ClPIN5d*-F | AGTGAAAACCTCCTCCTTACGC | Determination of *ClPIN5d* gene expression |
| *ClPIN5d*-R | CGTGTTCTCCTTCAGGCTTTTG |  |
| *ClPIN6*-F | TTATGCGATGTTGGCACCTC | Determination of *ClPIN6* gene expression |
| *ClPIN6*-R | ACAAGCAAGGGAACATGTGC |  |

(continued table)

| Primer names | Primer sequence（5’—3’） | Note |
| --- | --- | --- |
| *ClPIN7a*-F | ACGCTGAAATTGGCAACGAC | Determination of *ClPIN7a* gene expression |
| *ClPIN7a*-R | AGTGAACAAGGCCCTAACGAC |  |
| *ClPIN7b*-F | AAGGGTTGCAGTCGTCAAAG | Determination of *ClPIN7b* gene expression |
| *ClPIN7b*-R | AATCCGTTGGCCCCTTTTAC |  |
| *ClPIN8*-F | AGGAGGCAAGAGACAGAAATGG | Determination of *ClPIN8* gene expression |
| *ClPIN8*-R | TGGGGTTGATGATGAGCTTCC |  |
