# Supplementary figures and images for: PIN1a-mediated auxin release from rootstock cotyledon contributes to healing in watermelon as revealed by seeds soaking-VIGS and cotyledon grafting
Source: Hortic Res. 2024 Nov 26;12(3):uhae329. doi: 10.1093/hr/uhae329 (PMC11883227; doi:10.1093/hr/uhae329)

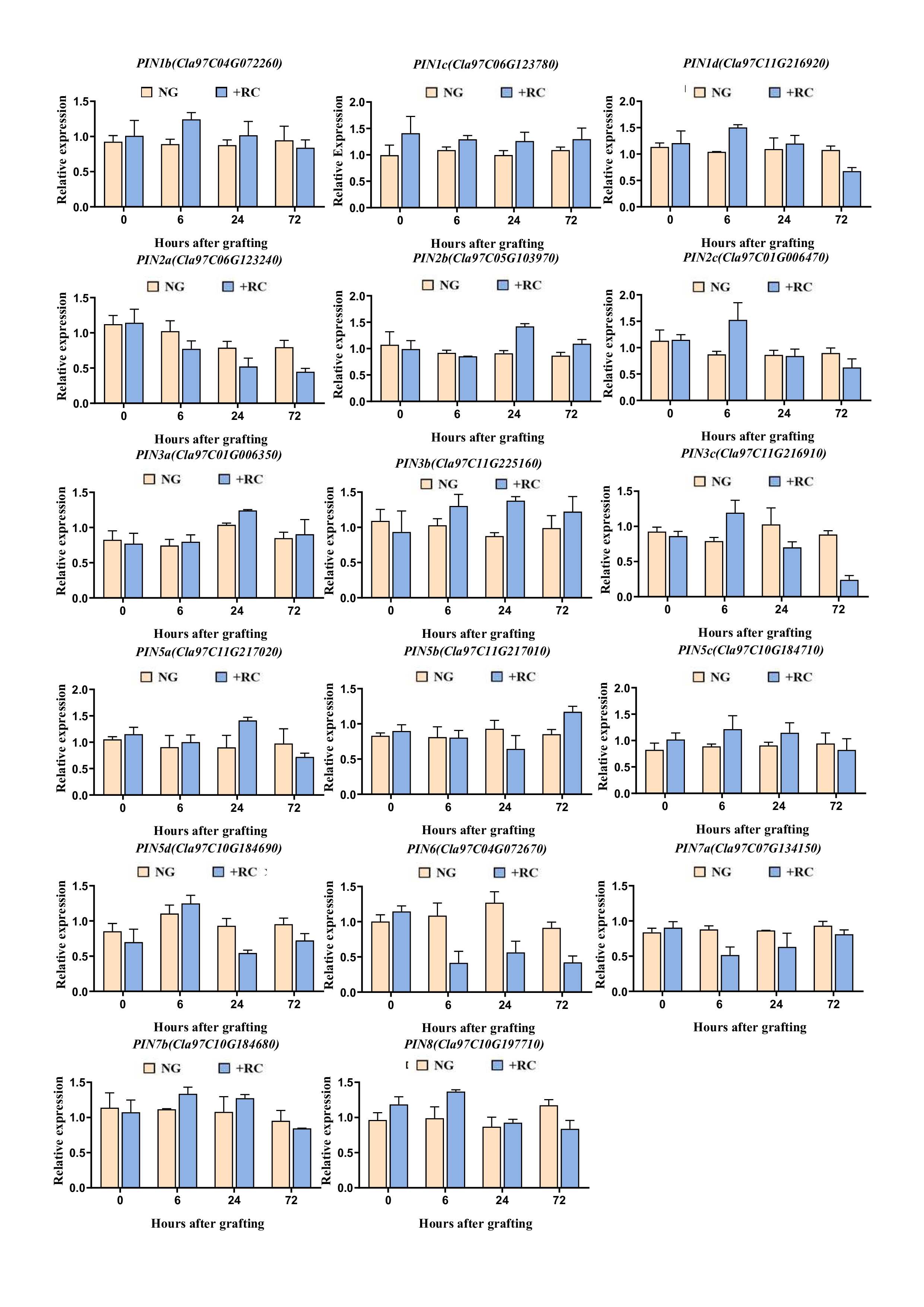

Supplement: Web_Material_uhae329 [file web_material_uhae329.zip › Figure S1.jpg]

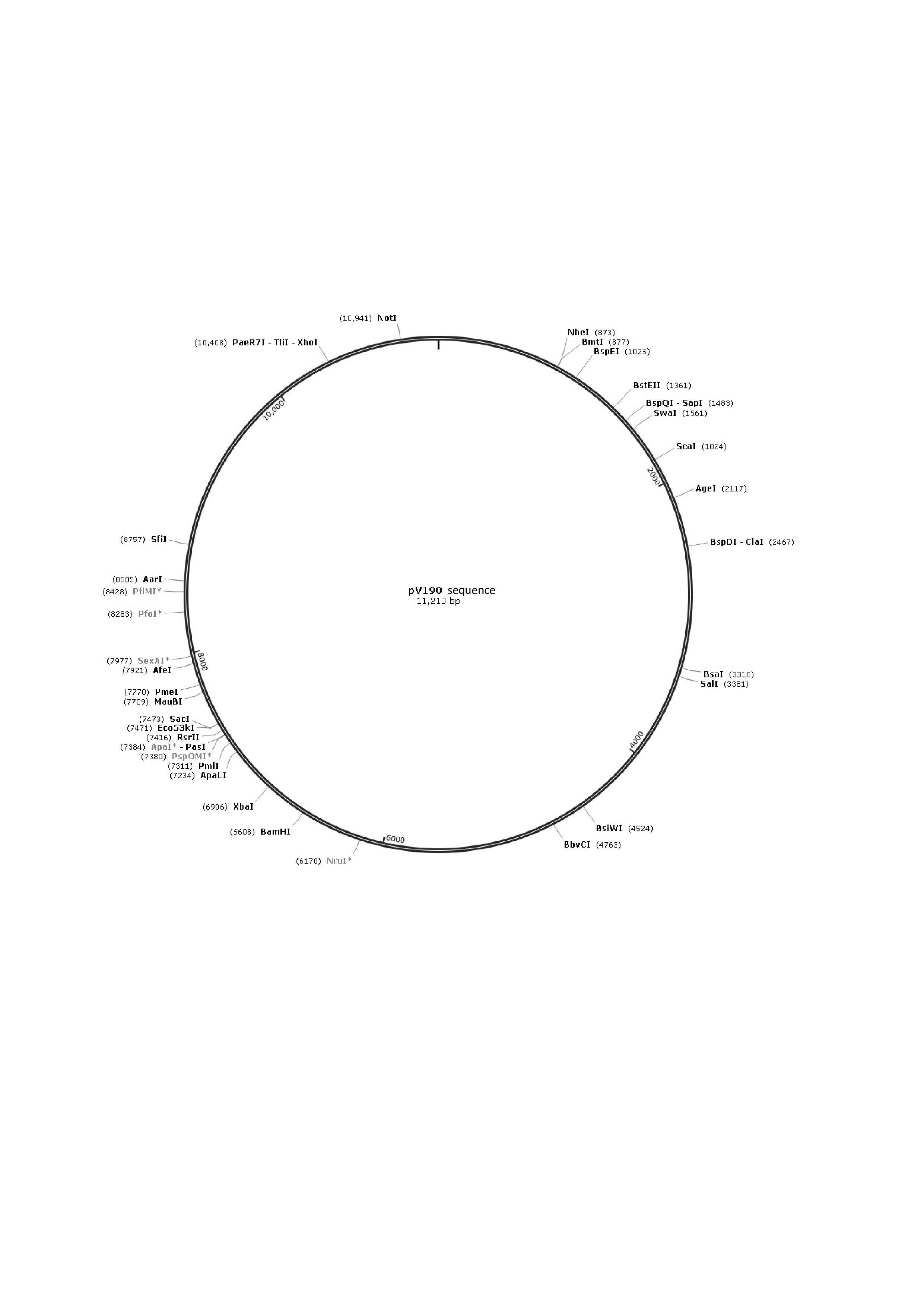

Supplement: Web_Material_uhae329 [file web_material_uhae329.zip › Figure S2.jpg]

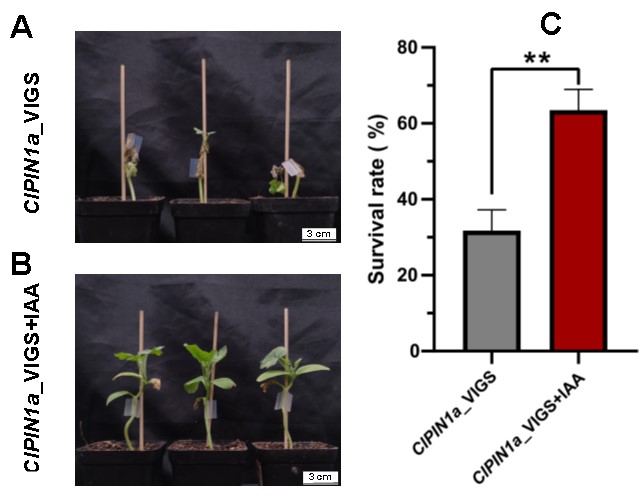

Supplement: Web_Material_uhae329 [file web_material_uhae329.zip › Figure S3.jpg]
